# Laparoscopic Versus Open Hemihepatectomy: The ORANGE II PLUS Multicenter Randomized Controlled Trial

**DOI:** 10.1200/JCO.23.01019

**Published:** 2024-04-19

**Authors:** Robert S. Fichtinger, Luca A. Aldrighetti, Mohammed Abu Hilal, Roberto I. Troisi, Robert P. Sutcliffe, Marc G. Besselink, Somaiah Aroori, Krishna V. Menon, Bjørn Edwin, Mathieu D'Hondt, Valerio Lucidi, Tom F. Ulmer, Rafael Díaz-Nieto, Zahir Soonawalla, Steve White, Gregory Sergeant, Bram Olij, Francesca Ratti, Christoph Kuemmerli, Vincenzo Scuderi, Frederik Berrevoet, Aude Vanlander, Ravi Marudanayagam, Pieter Tanis, Maxime J.L. Dewulf, Cornelis H.C. Dejong, Zina Eminton, Merel L. Kimman, Lloyd Brandts, Ulf P. Neumann, Åsmund A. Fretland, Siân A. Pugh, Gerard J.P. van Breukelen, John N. Primrose, Ronald M. van Dam, ORANGE II PLUS Collaborative

**Affiliations:** ^1^ Department of Surgery, Maastricht University Medical Center+, Maastricht, the Netherlands; ^2^ Department of Surgery and Transplantation, University Hospital RWTH Aachen, Aachen, Germany; ^3^ Hepatobiliary Surgery Division, IRCCS San Raffaele Hospital, Milan, Italy; ^4^ Department of Surgery, Southampton University Hospital NHS Foundation Trust, Southampton, United Kingdom; ^5^ Department of Surgery, Poliambulanza Hospital, Brescia, Italy; ^6^ Division of HPB, Minimally Invasive and Robotic Surgery, Department of Clinical Medicine and Surgery, Transplantation Service, Federico II University, Naples, Italy; ^7^ Department of General, Hepatobiliary and Liver Transplantation Surgery, Ghent University Hospital, Ghent, Belgium; ^8^ Department of Surgery, University Hospitals Birmingham NHS Foundation Trust, Birmingham, United Kingdom; ^9^ Department of Surgery, Amsterdam UMC, Location University of Amsterdam, Amsterdam, the Netherlands; ^10^ Cancer Center Amsterdam, the Netherlands; ^11^ Department of Surgery, Plymouth Hospitals NHS Trust, Plymouth, United Kingdom; ^12^ Department of Surgery, King's College Hospital NHS Foundation Trust, London, United Kingdom; ^13^ Intervention Center and Department of Hepatic, Pancreatic and Biliary Surgery, Oslo University Hospital and Institute of Medicine, University of Oslo, Oslo, Norway; ^14^ Department of Digestive and Hepatobiliary/Pancreatic Surgery, AZ Groeninge, Kortrijk, Belgium; ^15^ Department of Digestive Surgery, Unit of Hepatobiliary Surgery and Transplantation, Hôpitaux Universitaires de Bruxelles, Hôpital Erasme, Brussels, Belgium; ^16^ Department of Hepato-Biliary Surgery, Aintree University Hospital NHS Foundation Trust, Liverpool, United Kingdom; ^17^ Department of Surgery, Oxford University Hospitals NHS Foundation Trust, Oxford, United Kingdom; ^18^ Department of Surgery, Newcastle Upon Tyne Hospitals NHS Foundation Trust, Newcastle Upon Tyne, United Kingdom; ^19^ Department of Digestive and Hepatobiliary/Pancreatic Surgery, Jessa Hospital, Hasselt, Belgium; ^20^ GROW—School for Oncology and Reproduction, Maastricht University, Maastricht, the Netherlands; ^21^ Department of Surgery, Free University Hospital, AZ Jette Hospital, Brussels, Belgium; ^22^ Southampton Clinical Trials Unit, University of Southampton, Southampton, United Kingdom; ^23^ Department of Clinical Epidemiology and Medical Technology Assessment, Maastricht University Medical Center+, Maastricht, the Netherlands; ^24^ Department of Surgery, University Hospital Essen, Essen, Germany; ^25^ Department of Oncology, Addenbrooke's Hospital, Cambridge, United Kingdom; ^26^ Department of Methodology and Statistics, CAPHRI Care and Public Health Research Institute Maastricht University, Maastricht, the Netherlands; ^†^ Deceased

## Abstract

**PURPOSE:**

To compare outcomes after laparoscopic versus open major liver resection (hemihepatectomy) mainly for primary or metastatic cancer. The primary outcome measure was time to functional recovery. Secondary outcomes included morbidity, quality of life (QoL), and for those with cancer, resection margin status and time to adjuvant systemic therapy.

**PATIENTS AND METHODS:**

This was a multicenter, randomized controlled, patient-blinded, superiority trial on adult patients undergoing hemihepatectomy. Patients were recruited from 16 hospitals in Europe between November 2013 and December 2018.

**RESULTS:**

Of the 352 randomly assigned patients, 332 patients (94.3%) underwent surgery (laparoscopic, n = 166 and open, n = 166) and comprised the analysis population. The median time to functional recovery was 4 days (IQR, 3-5; range, 1-30) for laparoscopic hemihepatectomy versus 5 days (IQR, 4-6; range, 1-33) for open hemihepatectomy (difference, –17.5% [96% CI, –25.6 to –8.4]; *P* < .001). There was no difference in major complications (laparoscopic 24/166 [14.5%] *v* open 28/166 [16.9%]; odds ratio [OR], 0.84; *P* = .58). Regarding QoL, both global health status (difference, 3.2 points; *P* < .001) and body image (difference, 0.9 points; *P* < .001) scored significantly higher in the laparoscopic group. For the 281 (84.6%) patients with cancer, R0 resection margin status was similar (laparoscopic 106 [77.9%] *v* open 122 patients [84.1%], OR, 0.60; *P* = .14) with a shorter time to adjuvant systemic therapy in the laparoscopic group (46.5 days *v* 62.8 days, hazard ratio, 2.20; *P* = .009).

**CONCLUSION:**

Among patients undergoing hemihepatectomy, the laparoscopic approach resulted in a shorter time to functional recovery compared with open surgery. In addition, it was associated with a better QoL, and in patients with cancer, a shorter time to adjuvant systemic therapy with no adverse impact on cancer outcomes observed.

## INTRODUCTION

Surgical resection of the liver is central to the curative treatment strategy of several cancers including colorectal liver metastases, hepatocellular carcinoma, and cholangiocarcinoma. Favorable long-term outcomes are achieved in up to a quarter of patients, provided a complete resection can be accomplished.^1-3^ Optimizing postoperative recovery is essential not only for the quality of life (QoL) of patients but also to delivery of further oncological treatments when indicated.^4^

**CONTEXT**

**Key Objective**
Liver resection is a key treatment in the curative management of primary and metastatic hepatic malignancy. Randomized studies have confirmed the benefit of laparoscopic (minimally invasive) surgery in small resections of the liver, but there is no level one evidence supporting the use of laparoscopic major hepatectomy, which is technically more complex.
**Knowledge Generated**
This study provides evidence that laparoscopic hemihepatectomy is superior to open hemihepatectomy in terms of time functional recovery, postoperative quality of life, time to adjuvant systemic therapy when given, and cost-effectiveness. The oncological efficacy appears similar.
**Relevance *(E.M. O'Reilly)***
This phase III trial adds to the body of evidence supporting a minimally invasive surgical approach over open surgery for major liver resections across a spectrum of primary and metastatic malignancies and with maintenance of oncologic outcomes.*
*Relevance section written by *JCO* Associate Editor Eileen M. O'Reilly, MD.

Minimally invasive surgery, such as laparoscopy, reduces the physical impact of surgery, accelerates postoperative recovery, and because of the decreased inflammatory response may improve cancer outcomes.^4,5^ Alongside the laparoscopic approach, the increased use of enhanced recovery after surgery protocols in hepatobiliary surgery has contributed to reduced length of hospital stay, postoperative complications, and overall hospital costs while preserving patient safety.^6^

Laparoscopic surgery is now established as standard of care for minor liver resections.^7-10^ Resection of the right or left side of the liver, so called hemihepatectomy, is considered a major liver resection. The technical complexity of hemihepatectomy is such that it is more challenging to perform using laparoscopic techniques. Furthermore, these operations carry a higher complication rate because of the volume of liver that needs to be resected, a larger wound surface, longer time in anesthesia, and exposure of major vessels and bile ducts.^11-14^ Experience is growing, but its adoption has appropriately been limited by the absence of level one evidence supporting its use.^15^

The ORANGE II PLUS trial sought to assess whether the laparoscopic approach to hemihepatectomy improves clinical and oncological outcomes for patients compared with open surgery. To standardize perioperative management and optimize recovery across both groups of the trial, all patients were managed within an enhanced recovery after surgery pathway.^16^

## PATIENTS AND METHODS

### Patients

Eligible patients were adults age 18 years or older, with a BMI between 18 and 35 kg/m^2^, an American Society of Anesthesiologists status of <IV, and an indication for a left or right hemihepatectomy, suitable for both laparoscopic and open approach as decided at the local multidisciplinary tumor board meeting. One additional ablation or metastasectomy in the remaining liver remnant was permitted.

The following patients were excluded: those who were pregnant or breastfeeding, any previous hepatectomy, or any hepatic lesions too close to central vascular or biliary structures. Previous open abdominal surgery and systemic anticancer therapy were not considered contraindications for inclusion. Detailed eligibility criteria are shown in the Data Supplement (Table S1, online only).

Patients were recruited from 16 centers in Europe. Ethical approval was obtained from the institutional review board of each participating center, and data were reviewed by an independent Data and Safety Monitoring Board (Data Supplement). Written informed consent was obtained from all patients before random assignment.

### Random Assignment and Masking

Patients were randomly assigned in a 1:1 ratio to laparoscopic or open hemihepatectomy using online random assignment software (TENALEA, Version 3.0). A minimization scheme was used to balance patient allocation, with stratification by center and side of hemihepatectomy.^17^ In the case of an imbalance of two patients, the probability of being assigned to the underrepresented group was 90%.

Patients and ward personnel were masked to treatment allocation using a large abdominal dressing that covered all surgical incisions, proven effective in two previous randomized trials (Data Supplement, Fig S1). This dressing remained in place until postoperative day 4, unless patients had achieved functional recovery sooner or if the patient's clinical condition necessitated unblinding.^18,19^

### Procedures

All participating centers were experienced in laparoscopic and open hemihepatectomy (Data Supplement, Table S2). At the start of trial accrual, four centers had performed more than 40 laparoscopic hemihepatectomies, and 12 centers had performed between 10 and 40. All centers had a standardized perioperative enhanced recovery program in place.^16,20^

For pragmatic reasons and to preserve external validity, the surgical techniques were not standardized. Participating surgeons could use their preferred methods for abdominal access, liver parenchymal transection, vascular control, and closure of the surgical wound.

### Data Collection and Outcomes Measures

The primary end point was time to functional recovery, defined as the time in days between the end of surgery and the time point the patient met five predefined criteria, as observed and scored by the blinded ward personnel or trial nurse. The five criteria were adequate pain control with oral analgesia alone, independent mobility (mobility score of ≥8 or at the preoperative level),^18,21^ tolerance of solid food ≥24 hours, normalized or improving liver function tests (total bilirubin, ALT, and/or AST) and blood clotting (international normalized ratio), and independence from intravenous fluid administration. ^22^

Secondary end points were length of hospital stay, intraoperative blood loss, operating time, intraoperative incidents, conversion rate from laparoscopic to open surgery, in-hospital and 90-day mortality, 90-day (liver specific) morbidity, readmission, health-related QoL, and costs. Postoperative complications were divided into minor (Clavien-Dindo grade 1 and 2), major (Clavien-Dindo grade ≥3a), and cumulative in accordance with the Comprehensive Complication Index.^23,24^ In addition, the following oncological end points (where appropriate) were included: resection margin status, time to adjuvant systemic therapy initiation when delivered, disease-free survival (DFS), and overall survival. Overall survival was defined as the time from surgery to death from any cause. DFS was defined as the time from surgery to death from any cause or recurrence of cancer, whichever occurred first. Liver-specific morbidity was defined as the occurrence of one or more of the following complications: operative mortality, intra-abdominal hemorrhage, ascites, bile leakage, intra-abdominal abscess, or postoperative liver failure.^25^ Intraoperative and postoperative costs were estimated on the basis of clinician-reported individual-level resource use (Data Supplement). Cost-effectiveness was expressed by the incremental cost per quality-adjusted life year (QALY) gained. Health-related QoL was measured using the EuroQoL EQ-5D-3L and the European Organization for Research and Treatment of Cancer (EORTC) QoL Questionnaire C30.^26,27^ Body image was assessed using a body image questionnaire.^28^

### Sample Size

Anticipating a drop-out rate of 10% and a loss in df for estimating covariate effects (hemihepatectomy side and center), a total sample size of 250 patients was planned to demonstrate a 2-day reduction in time to functional recovery with a two-sided 4% level of significance and a power of 80%, assuming a standard deviation (SD) of time to functional recovery of 5 days within both groups.^29,30^ A two-sided 4% level of significance was used instead of 5% to compensate for the planned interim analysis halfway through the trial with a two-sided 1% level of significance, thus preserving an overall type I error rate of 5%.^31^

### Statistical Analyses

Before the trial started, there were no data available on time to functional recovery, so length of hospital stay was used to estimate the effect size. Because of an unforeseen sample size extension, additional analyses were performed to crosscheck for any influence (Data Supplement).^47^

The primary analysis was by intention-to-treat. Patients were excluded if they had withdrawn their consent or not undergone surgery. An additional per-protocol analysis excluded five patients who underwent surgery but not hemihepatectomy. Procedures converted to hand-assisted or open surgery from laparoscopy remained in the laparoscopic group for all analyses.

Time to functional recovery was analyzed with fixed and mixed linear regression on treatment group, adjusting for center (dummy coded), hemihepatectomy side (left/right), age (years, continuous), sex (male/female), and tumor type (benign/malignant) at the two-sided 4% significance level. The secondary surgical and oncological end points were assessed with mixed regression (with center as random effect), linear for continuous outcomes, logistic for binary outcomes, and Cox for time-to-event outcomes, all at a two-sided 1% significance level in view of the multiple outcome testing. Cost and cost-effectiveness data were analyzed using nonparametric bootstrapping techniques.

Subgroup analyses were performed to assess the outcome difference between the treatment groups on the basis of the covariates used in the regression models and also on the basis of various other predefined preoperative and intraoperative covariates. These subgroup analyses were only exploratory unless significant interaction was found between treatment groups and the covariate at hand.

Analyses were performed using SPSS Statistics software (IBM, Windows Version 27.0.1.0) and R software (R project for Statistical Computing, Windows Version 4.1.0).

## RESULTS

**FIG 1.**
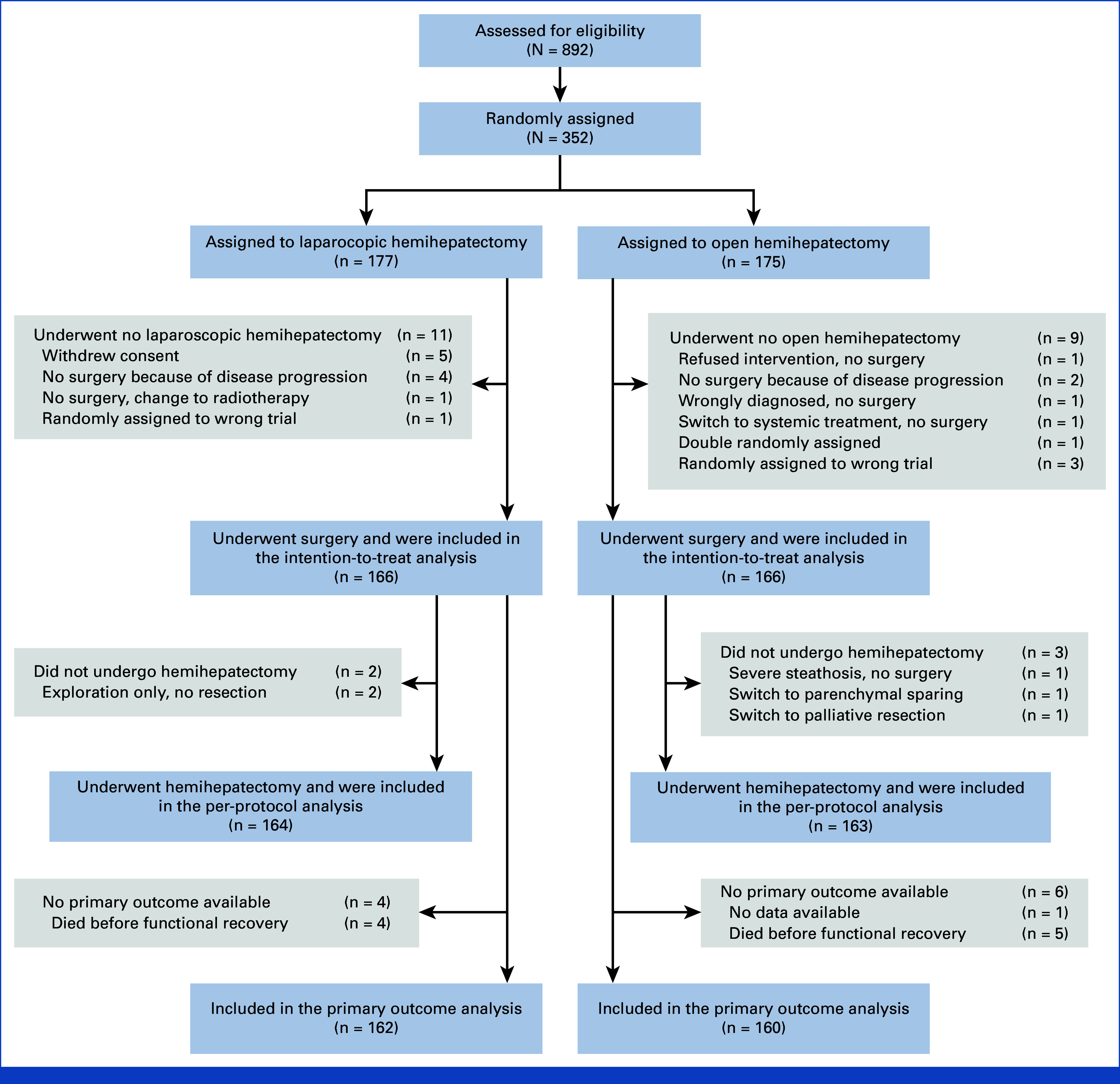
CONSORT diagram of the ORANGE II PLUS trial. Surgical procedures that were converted from laparoscopy to hand-assisted or open surgery were considered a laparoscopic procedure in both the intention-to-treat analysis and the per-protocol analysis.

Between November 2013 and December 2018, 829 patients were screened and 352 were randomly assigned. The median time from random assignment to surgery was 7.5 days (IQR, 2-22; range, 0-83) in the laparoscopic group and 9 days (IQR, 2-20; range, 0-178) in the open group. The intention-to-treat analysis included 332 patients. Figure 1 and the Data Supplement (Table S3) describe the study flow and reasons for withdrawal.

Clinical characteristics were well-balanced between treatment groups, Table 1. Right hemihepatectomy was the most common procedure, performed on 108 of 166 patients (65%) in the laparoscopic group and 105 of 166 patients (63%) in the open group. The majority underwent surgery for cancer (136 patients [48%] in the laparoscopic group and 145 patients [52%] in the open group) of whom most had colorectal liver metastases (165/281 patients [59%]). Neoadjuvant systemic therapy was administered to 58 of 136 patients (43%) in the laparoscopic group and to 58 of 145 patients (40%) in the open group (Data Supplement, Tables S4 and S5).

**TABLE 1.** Baseline Demographic and Clinical Characteristics of Patients Who Underwent Laparoscopic or Open Surgery

Characteristic	Surgery, No./Total (%)^a^	
	Laparoscopic (n = 166)	Open (n = 166)
Sex		
Female	67/166 (40)	70/166 (42)
Male	99/166 (60)	96/166 (58)
Age at surgery, mean (SD), years	61.5 (13.5)	62.6 (13.0)
BMI, median (IQR; range), kg/m^2^	26.0 (23-29; 17-37)	25.0 (22 to 28; 14 to 36)
Association of Anesthesiologists Classification		
I: Healthy	13/166 (8)	19/166 (11)
II: Mild systemic disease	93/166 (56)	91/166 (55)
III: Severe systemic disease	52/166 (31)	52/166 (31)
Missing	8/166 (5)	4/166 (5)
Eastern Cooperative Oncology Group performance status		
0: Asymptomatic, normal activity	121/166 (73)	123/166 (74)
1: Symptomatic, normal activity	36/166 (22)	40/166 (24)
2: Symptomatic, <50% bedridden	4/166 (2)	1/166 (1)
3: Symptomatic, >50% bedridden	1/166 (1)	0/166 (0)
4: 100% bedridden	0/166 (0)	0/166 (0)
Missing	4/166 (2)	1/166 (1)
Charlson Comorbidity Index, mean (SD), points	6.3 (3.2)	6.2 (2.8)
Previous abdominal surgery	87/166 (52)	92/166 (55)
Preoperative portal vein embolization	16/166 (10)	9/166 (5)
Neoadjuvant systemic therapy	58/136 (43)	58/145 (40)
Radiological diagnosis		
Benign	25/166 (15)	20/166 (12)
Hemangioma	6/166 (4)	6/166 (4)
Adenoma	5/166 (3)	0/166 (0)
Follicular nodular hyperplasia	0/166 (0)	2/166 (1)
Other benign	14/166 (8)	12/166 (7)
Cancer	141/166 (85)	146/166 (88)
Colorectal metastasis	90/166 (54)	78/166 (48)
Hepatocellular carcinoma	22/166 (13)	25/166 (15)
Cholangiocarcinoma	17/166 (10)	30/166 (18)
Other malignant	12/166 (7)	13/166 (7)
Hemihepatectomy side		
Left	61/166 (37)	58/166 (35)
Right	105/166 (63)	108/166 (65)
Additional contralateral surgery		
Wedge resection	18/166 (10)	18/166 (10)
Ablation	6/166 (3)	3/166 (2)
Ablation and wedge resection	2/166 (1)	2/166 (1)

Abbreviation: SD, standard deviation.

^a^ Data are reported as No./total (%) unless otherwise indicated. Percentages may not total 100 because of rounding.

The mean time to functional recovery was 4.7 days (SD, 3.5) in the laparoscopic group and 5.9 days (SD, 4.4) in the open group. In view of the non-normal distribution of the primary outcome, the median time to functional recovery is primarily reported: 4 days (IQR, 3-5; range, 1-30) in the laparoscopic group and 5 days (IQR, 4-6; range, 1-33) in the open group (Table 2, Data Supplement, Table S6). The median time taken to achieve the individual components of the end point is depicted in the Data Supplement (Fig S2).

**TABLE 2.** Primary and Secondary Outcomes of Participants Who Underwent Surgery in the Intention-To-Treat Analysis, Multivariable Model

Primary End Point	Laparoscopic (n = 162)	Open (n = 160)	Model 2—Multivariable^b^	
			% Difference/β (96% CI)	*P*
Functional recovery, median, days^a^ (IQR; range)	4 (3-5; 1-30)	5 (4-6; 1-33)	–17.5 (–25.6 to –8.4)	<.001

Secondary End Point—Surgical^c^	Laparoscopic (n = 166)	Open (n = 166)	Model 2—Multivariable^b^			
			% Difference/β (99% CI)	Odds Ratio (99% CI)	Hazard Ratio (99% CI)	*P*
Hospital stay, median, days (IQR; range)	5 (4-7; 1-43)	6 (5-7; 2-50)	–16.4 (–27.7 to –3.9)			.002
Blood loss, median, mL (IQR; range)	450 (300-775; 0-5,000)	450 (300-785; 50-16,000)				.79^d^
Operation time median, minutes (IQR; range)	310 (255-379; 45-595)	254 (194-301; 41-604)				<.001^d^
Conversions to hand-assisted surgery	2 (1.2)	NA				
Conversions to open surgery	26 (15.7)	NA				
Intraoperative inotropy use	73 (47.4)	67 (40.4)		1.60 (0.74 to 3.45)		.12
Satava 1 intraoperative incidents	13 (7.8)	25 (15.1)		0.55 (0.21 to 1.46)		.12
Satava 2 intraoperative incidents	5 (3)	5 (3)		1.10 (0.19 to 6.39)		.89
Satava 3 intraoperative incidents	0 (0)	2 (1.2)				
Comprehensive Complication Index	—	—	–1.30 (–7.13 to 4.52)			.56
CCI >0	73 (44)	79 (47.6)		0.86 (0.47 to 1.60)		.54
CCI when excluding grade 1	49 (29.5)	56 (33.7)		3.46 (-8.29 to 15.21)		.44
Minor complications (grade 1 or 2)	49 (29.5)	51 (30.7)		0.96 (0.50 to 1.81)		.85
Major complications (≥grade 3A)	24 (14.5)	28 (16.9)		0.84 (0.37 to 1.89)		.58
Prolonged admission (>10 days)	16 (9.6)	24 (14.5)		0.70 (0.29 to 1.69)		.29
30-day readmission	13 (7.8)	12 (7.2)		1.12 (0.37 to 3.38)		.79
90-day mortality	5 (3)	5 (3)		1.02 (0.27 to 3.92)		.97
90-day morbidity	73 (44)	79 (47.6)				
90-day liver specific morbidity	23 (13.9)	26 (15.7)		0.89 (0.40 to 2.00)		.71
90-day readmission	22 (13.3)	20 (12)		1.12 (0.46 to 2.74)		.74
Global health status^e^			3.19 (0.71 to 5.68)			<.001
Body image^e^			0.86 (0.46 to 1.26)			<.001
Costs, mean, USD (99% BCI)	17,140 (16,223 to 18,240)	15,478 (14,203 to 16,886)	1,662 (98 to 3,334)			
Quality-adjusted life years, observed mean (SD)	0.83 (0.22)	0.80 (0.24)	0.05 (–0.003 to 0.10)			.080
Incremental cost-effectiveness ratio, USD	33,119					

Secondary End Point—Oncological^f^	Laparoscopic (n = 136)	Open (n = 145)	Model 2—Multivariable^b^			
			% Difference (99% CI)	Odds Ratio (99% CI)	Hazard Ratio (99% CI)	*P*
Time to adjuvant systemic therapy, median, days^g^ (IQR; range)	46.5 (36.5-62.8; 6-84)	62 (47-72; 22-88)			2.20 (1.01 to 4.77)	.009
R0 resection margin	106/132 (77.9)	122/140 (84.1)		0.60 (0.25 to 1.45)		.14
R1 and R2 resection margin	26/132 (19.1)	18/140 (12.4)		1.65 (0.69 to 3.97)		.14
Recurrence total	66 (48.5)	84 (57.9)		0.72 (0.38 to 1.37)		.19
Recurrence liver only	36 (26.5)	50 (34.5)		0.67 (0.34 to 1.34)		.13
Disease-free survival^h^	55 (40.7)	51 (35.4)				.46
Overall survival^h^	67 (57.3)	86 (65.6)				.59

Abbreviations: BCI, bootstrapped confidence interval; CCI, comprehensive complication index; NA, not applicable; USD, US dollars.

^a^ Time to functional recovery could not be determined for four patients in the laparoscopic group and six patients in the open group.

^b^ Result adjusted for sex, age, hemihepatectomy side, benign/malignant tumor type, and treatment center. In all analyses, the open group is used as reference group.

^c^ Data are reported as No./total (%) unless otherwise indicated. Percentages may not total 100 because of rounding.

^d^ Mann-Whitney *U* test.

^e^ Points difference over 12 months after surgery. Result adjusted for sex, age, hemihepatectomy side, benign/malignant tumor type, treatment center, and baseline difference.

^f^ Patients with malignant disease only.

^g^ Adjuvant systemic therapy was given to 38 patients in the laparoscopic group and 31 patients in the open group.

^h^ Log-rank test.

The fixed-effect regression analyses of the log-transformed time to functional recovery showed that time to functional recovery was significantly shorter in the laparoscopic group (difference –17.5% [96% CI, 25.6 to –8.4]; naïve method *P* < .001 and combinatory method *P* = .004). These results were also confirmed with nonparametric tests (naïve method *P* < .001 and combinatory method *P* = .026). For further details on the regression analyses, see the Data Supplement. Outcomes were similar for the per-protocol analysis (Data Supplement Tables S7 and S8).

Median length of hospital stay was shorter in the laparoscopic group (5 days [IQR, 4-7; range, 1-43] *v* 6 days [IQR, 5-7; range, 2-50] difference, –16.4% [99% CI, –27.7 to –3.9]; *P* = .002) while the duration of surgery was longer (310 minutes, IQR, 255-379; range, 45-595) versus 254 minutes (IQR, 194-301; range, 41-604; *P* < .001). Median overall blood loss was comparable, *P* = .79 (Table 2).

For 28 patients (17%), the laparoscopic resection was converted to an open procedure. Of these patients, seven (25%) were converted for urgent reasons (mainly bleeding) and 21 (75%) for nonurgent reasons (predominantly uncertainty concerning resection margins). The median time to functional recovery in those 28 patients converted to an open procedure was 5 days (IQR, 4-6; range, 1-9, Data Supplement, Table S9).

The overall incidence of adverse events was similar between the groups (Table 2). Major complications occurred in 24 patients (15%) in the laparoscopic group and in 28 patients (17%) in the open group (odds ratio [OR], 0.84 [99% CI, 0.37 to 1.89]; *P* = .58). There were five deaths (3%) in the laparoscopic group and five (3%) in the open group within 90 days of surgery (OR, 1.02 [99% CI, 0.27 to 3.9]; *P* = .97). One death in the laparoscopic group was due to disease progression. A detailed description of the most common complications is in the Data Supplement (Tables S10 and S11).

### Secondary Outcomes: QoL, Body Image, Costs, and Cost-Effectiveness

**FIG 2.**
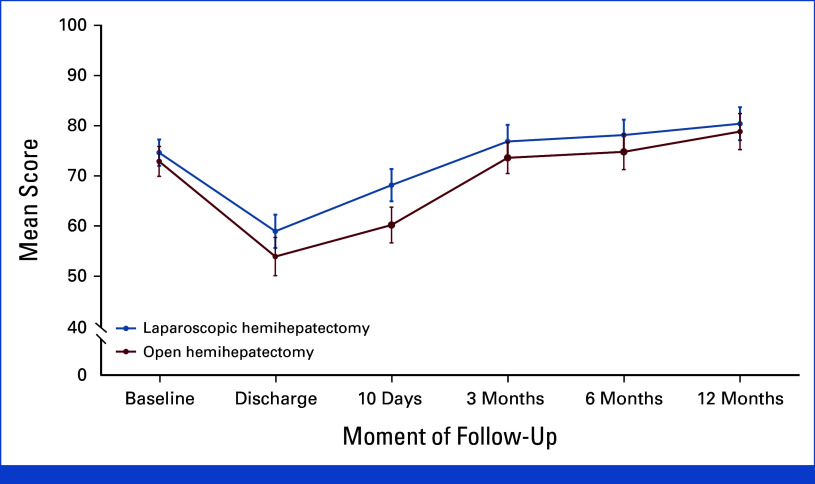
Global health status of the European Organization for Research and Treatment of cancer—Quality-of-Life Questionnaire C30.

Over the first year after laparoscopic hemihepatectomy, Global Health Status (derived from EORTC QoL questionnaires) was significantly better in the laparoscopic group (difference, 3.19 points [99% CI, 0.71 to 5.68]; *P* < .001) (Table 2, Fig 2). In addition, patients in the laparoscopic group reported significantly less deterioration of satisfaction with body image compared with the open group (difference, –0.86 points [99% CI, –1.26 to –0.46]; *P* < .001).

Intraoperative and postoperative costs per patient were higher for laparoscopic hemihepatectomy (mean difference, $1,662 in US dollars [USD] [99% CI, $98 (USD) to $3,334 (USD)]). However, the laparoscopic group gained an additional 0.05 QALYs in 12 months compared with the open group. As a result, the incremental cost-effectiveness ratio was $33,119 (USD) per additional QALY (Table 2). On the basis of the available evidence from the trial and using the Dutch-based maximum willingness-to-pay threshold of $72,240 (USD) (ie, €80,000), laparoscopic hemihepatectomy has a 77% probability to be a cost-effective alternative to open surgery. The cost-effectiveness acceptability curve (Data Supplement, Fig S3) shows the decision uncertainty in relation to a range of willingness-to-pay thresholds.

### Secondary Outcomes: Oncological Results

Additional outcomes were obtained for the patients undergoing resection for cancer (laparoscopic: n = 136, 82%; open: n = 145, 87%). R0 resection margins (≥1 mm) were achieved for 106 patients (78%) in the laparoscopic group compared with 122 patients (84%) in the open group (OR, 0.60 [99% CI, 0.25 to 1.45]; *P* = .14, Data Supplement, Table S12). For those who received adjuvant systemic therapy, the time interval between surgery and initiation of treatment was significantly shorter in the laparoscopic group (46.5 days) compared with the open group (62.8 days; hazard ratio, 2.20 [99% CI, 1.01 to 4.77]; *P* = .009; Table 2). The use of systemic therapy was largely restricted to those patients with colorectal liver metastases and cholangiocarcinoma with some patients receiving treatment in the neoadjuvant setting before trial recruitment (Data Supplement, Tables S4 and S5).

**FIG 3.**
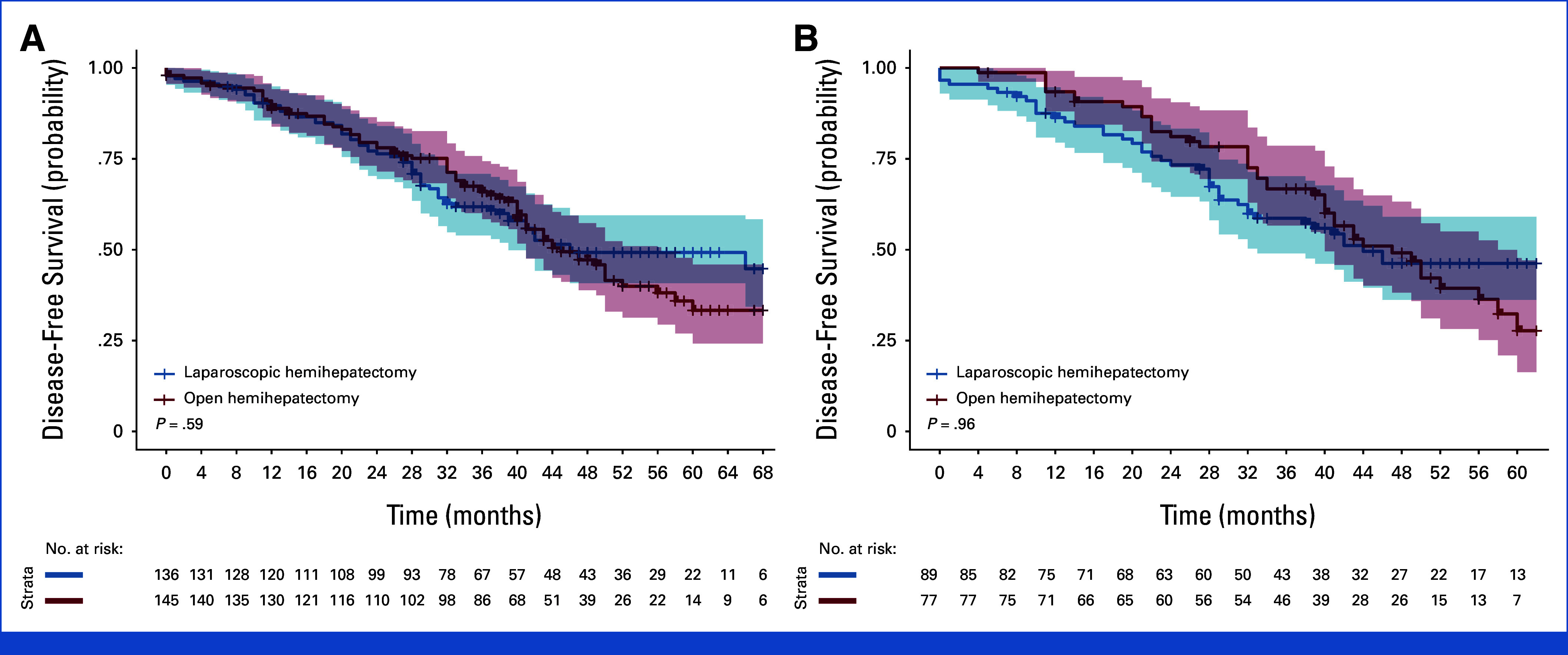
Kaplan-Meier curves of probability of disease-free survival for laparoscopic hemihepatectomy versus open hemihepatectomy, (A) curtailed at a maximum follow-up time of 68 months for all malignancies (follow-up index 65%) (B) and for colorectal liver metastases at a maximal follow-up time of 62 months (follow-up index 67%). 99% CI in shadings.

**FIG 4.**
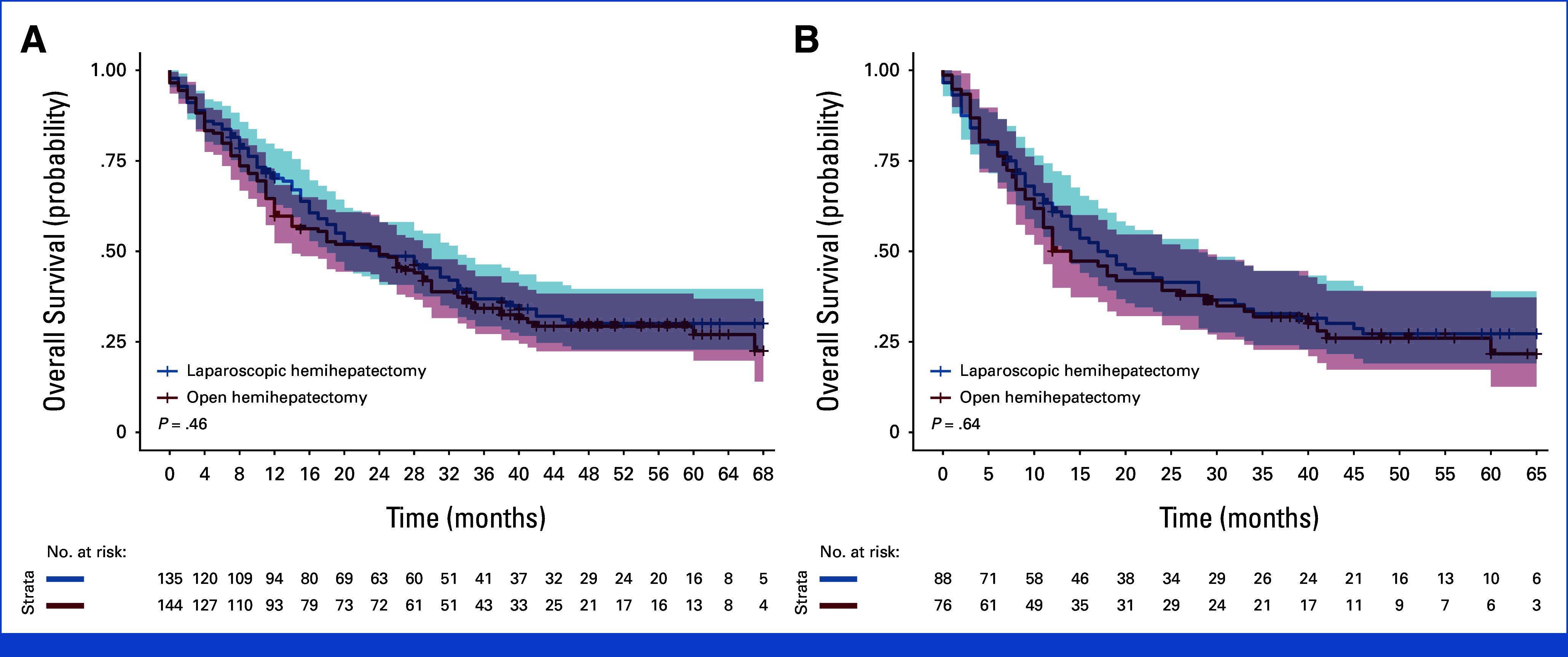
Kaplan-Meier curves of probability of overall survival for laparoscopic hemihepatectomy versus open hemihepatectomy, (A) curtailed at a maximum of 68 months for all malignancies (follow-up index 43%) and (B) for colorectal liver metastases at a maximum follow-up time of 65 months (follow-up index 39%). 99% CI in shadings.

Recurrence was diagnosed in 66 patients (49%) in the laparoscopic group and 84 patients (58%) in the open group (OR, 0.72 [99% CI, 0.38 to 1.37]; *P* = .19). Of them, 36 patients (27%) in the laparoscopic group and 50 patients (35%) in the open group had a recurrence in the liver (OR, 0.67 [99% CI, 0.34 to 1.34]; *P* = .13, Data Supplement, Table S13). At a median follow-up of 53 months (IQR, 39-63; range, 0-86), there were no significant differences in disease-free or overall survival between the groups (Figs 3 and 4).

The outcomes of the per-protocol analysis were similar for all secondary outcomes (Data Supplement, Tables S7 and S8).

### Subgroup Analyses

In the primary outcome analyses, interaction of treatment was tested with sex, age, hemihepatectomy side (left/right), surgical center, and tumor type (benign/malignant). A significant interaction was found for surgical center (*P* < .01 fixed regression, *P* < .05 mixed regression, Data Supplement). To determine whether this interaction might be due to differences in experience with laparoscopic surgery, an additional analysis was conducted in which centers with moderate experience (10-40 laparoscopic hemihepatectomies performed before the trial) were compared with centers with high experience (>40 laparoscopic hemihepatectomies). No interaction of the factor experience with treatment was found for time to functional recovery. Similarly, no significant interaction was found for time to functional recovery with any of the 10 predefined preoperative and intraoperative covariates (Data Supplement, Tables S14 and S15). The reduced time to functional recovery for the laparoscopic treatment group remained across all subgroups.

## DISCUSSION

To our knowledge, this is the first randomized clinical trial to evaluate the benefit of the laparoscopic approach in the context of a major liver resection. The results demonstrate a reduction in time to functional recovery, an improvement in QoL, and a shorter time to initiation of systemic therapy in favor of the laparoscopic approach.

Time to functional recovery was selected as the primary outcome measure because it avoids confounders that can affect length of stay.^32-34^ The observed 1 day difference could be regarded as small, but even in the group assigned to open surgery, time to functional recovery was just 5 days. The excellent outcomes achieved across both groups of the trial likely reflect the experience of the surgical centers and the benefits of modern enhanced recovery after surgery protocols.^6^ Indeed, it seems unlikely that further surgical advances, such as the use of robotic techniques, will be able to demonstrate an additional meaningful improvement.^35,36^

Concerns exist regarding the quality of cancer surgery performed laparoscopically.^37^ Reassuringly, there was no evidence of inferior oncological outcomes. Specifically, the R0 resection and recurrence rates, including liver only recurrence, were comparable in both groups. While the trial was not powered to assess the impact on survival, the outcomes are appropriate for the clinical cohort.^1,38,39^ Surgical morbidity and mortality were similarly as expected with no significant differences between the groups.^15,40^

Of further interest is that the time interval between surgery and start of adjuvant systemic therapy was shorter in the laparoscopic group. It is generally accepted that it is optimal to commence adjuvant treatment as soon as possible after surgery for a number of cancers albeit that the longer-term oncological impact of this to the ORANGE II PLUS cohort are unknown.^41-43^ The evidence for adjuvant systemic therapy in colorectal liver metastases is debated, both in terms of whether there is a need at all for systemic therapy and if so the optimal sequencing of treatment. In biliary tract cancer, there is a greater consensus but these patients comprised a smaller population within the trial.^44,45^

The value of patient-reported outcomes is increasingly recognized. A previous trial of patients undergoing resection of colorectal liver metastases demonstrated an improvement in QoL in favor of the laparoscopic approach up to 4 months after surgery.^10^ The current trial similarly demonstrated an improvement which extended to over 1 year after the operation. Body image and cosmesis scores were also significantly better in the laparoscopic group.

Not all end points were superior in the laparoscopic group. Operating times were longer, consistent with observational series but in contrast to the OSLO-COMET trial.^8^ This likely reflects the additional complexity of performing a hemihepatectomy and the need for conversion to an open procedure, which can be essential to avoid compromising oncological outcomes. The longer operating time and requirement for specialist equipment also resulted in higher operating costs. However, when the improvement in QoL is considered, reflected in QALY, the calculated incremental cost effectiveness ratio for the laparoscopic approach is likely to meet funding criteria across major western health care systems.

Trials evaluating surgical techniques need to be undertaken when sufficient experience in the technique has been developed, but before the new approach has been universally adopted in the absence of randomized data.^46^ To facilitate recruitment to ORANGE II PLUS, inclusion was not restricted to a particular disease. Although the majority of patients had colorectal liver metastases, it does limit the ability to explore oncological end points with precision, particularly within cancer subtypes.

In conclusion, this trial demonstrated excellent recovery times for patients undergoing hemihepatectomy managed within an enhanced recovery program. The laparoscopic approach resulted in an even shorter time to functional recovery together with being cost-effective and associated with a better QoL. In patients with cancer, surrogate oncological outcomes such as pathological resection status and sites of recurrence are reassuring in the absence of being able to assess an effect on overall survival. The shorter time to commencing adjuvant systemic therapy may reflect more subtle advantages to the laparoscopic approach in terms of recovery not captured by the primary end point. These results are directly applicable to the majority of patients with an indication for hemihepatectomy worldwide and may support a larger role for liver surgery within oncological treatment pathways. If experience is available, a laparoscopic approach can be considered for all patients undergoing hemihepatectomy.

## CLINICAL TRIAL INFORMATION

NCT01441856 (ORANGE II PLUS)

## APPENDIX

**TABLE A1.** ORANGE II PLUS Collaborative

Center	Name	Role
Aachen University Hospital, Aachen, Germany	Ulf Neumann	Principal investigator
	Florian Ulmer	Medical staff involved in patient care
	Finja Clausen	Research nurse
Aintree University Hospital NHS Foundation Trust, Aintree, United Kingdom	Rafael Díaz-Nieto	Principal investigator
	Michelle Linforth	Research nurse
Amsterdam University Medical Centers, Amsterdam, the Netherlands	Marc Besselink	Principal investigator
	Pieter Tanis	Medical staff involved in patient care
	Burak Gorçek	PhD candidate
	Marcel van der Poel	PhD candidate
University Hospitals Birmingham NHS Foundation Trust, Birmingham, United Kingdom	Robert Sutcliffe	Principal investigator
	Ravi Marudanayagam	Medical staff involved in patient care
	Diana Hull	Research nurse
	Penelope Rogers	Research nurse
Erasmus Hospital, Brussels, Belgium	Valerio Lucidi	Principal investigator
	Viviane van Laethem	Research nurse
Ghent University Hospital, Ghent, Belgium	Roberto Troisi	Principal investigator
	Frederik Berrevoet	Medical staff involved in patient care
	Vincenzo Scuderi	Medical staff involved in patient care
	Aude Vanlander	Medical staff involved in patient care
	Betsy van Loo	Research nurse, trial coordinator
	Kathleen Segers	Research nurse
Jessa Hospital, Hasselt, Belgium	Gregory Sergeant	Principal investigator
Groeninge General Hospital, Kortrijk, Belgium	Mathieu D'Hondt	Principal investigator
	Celine Demeyere	Research nurse
King's College Hospital NHS Foundation Trust, London, the United Kingdom	Krishna Menon	Principal investigator
	Ane Zamalloa	Research nurse
Maastricht University Medical Center+, Maastricht, the Netherlands	Ronald van Dam	Principal investigator, trial leader and main investigator
	Cornelis Dejong	Medical staff involved in patient care
	Maxime Dewulf	Medical staff involved in patient care
	Lloyd Brandts	Trial statistician
	Robert Fichtinger	PhD candidate, trial coordinator
	Bram Olij	PhD candidate
	Merel Kimman	Health economics and quality of life expert
	E.M. Wong-Lun-Hing	PhD candidate
Maastricht University, Maastricht, the Netherlands	Gerard van Breukelen	Trial statistician
San Raffaele Hospital, Milan, Italy	Luca Aldrighetti	Principal investigator
	Francesca Ratti	Medical staff involved in patient care
Newcastle upon Tyne Hospitals NHS Foundation Trust, Newcastle, the United Kingdom	Steve White	Principal investigator
	Stuart Robinson	Medical staff involved in patient care
	Caroline Brunton	Research nurse
Oslo University Hospital, Oslo, Norway	Björn Edwin	Principal investigator
	Åsmund Fretland	Medical staff involved in patient care
	Davit Aghayan	PhD candidate
Oxford University Hospitals NHS Foundation Trust, Oxford, the United Kingdom	Zahir Soonawalla	Principal investigator
	Katherine Gordon-Quayle	Research nurse
University Hospitals Plymouth NHS Foundation Trust, Plymouth, the United Kingdom	Somaiah Aroori	Principal investigator
	Tracy Ward	Research nurse
University Hospital Southampton NHS Foundation Trust, Southampton, the United Kingdom	Mohammed Abu Hilal	Principal investigator
	John Primrose	Medical staff involved in patient care
	Christoph Kümmerli	PhD candidate
	Elizabeth Wedge	Research nurse
Southampton Clinical Trials Unit, Southampton, the United Kingdom	Zina Eminton	Trial manager
